# Impact of hospital-acquired pneumonia on the Medicare program

**DOI:** 10.1017/ice.2023.221

**Published:** 2024-03

**Authors:** Dian L. Baker, Karen K. Giuliano, Mark Desmarais, Chantal Worzala, Annie Cloke, Lu Zawistowich

**Affiliations:** 1 School of Nursing, California State University, Sacramento, California; 2 Elaine Marieb Center for Nursing and Engineering Innovation, Amherst, Massachusetts; 3 The Moran Company, Arlington, Virginia; 4 Alazro Consulting, Takoma Park, Maryland; 5 CapView Strategies, Brooklyn, New York; 6 CapView Strategies, Washington, DC

## Abstract

**Objective:**

Patient safety organizations and researchers describe hospital-acquired pneumonia (HAP) as a largely preventable hospital-acquired infection that affects patient safety and quality of care. We provide evidence regarding the consequences of HAP among 2019 Medicare beneficiaries.

**Design:**

Retrospective case–control study.

**Patients:**

Calendar year 2019 Medicare beneficiaries with HAP during an initial hospitalization, defined by *International Classification of Diseases, Tenth Revision, Clinical Modification* (ICD-10-CM) coding on inpatient claims (n = 2,457). Beneficiaries with HAP were matched using diagnosis-related group (DRG) codes with beneficiaries who did not experience HAP (n = 2,457).

**Methods:**

The 2019 calendar year Medicare 5% Standard Analytic Files (SAF), for inpatient, outpatient, physician, and all postacute hospital settings. The case group (HAP) and control group (non-HAP) were matched on disease severity, age, sex, and race and were compared for hospital length of stay, costs, and mortality during the initial hospitalization and across settings for 30, 60, and 90 days after discharge. The 2019 fiscal year MedPAR Claims data were used to determine Medicare costs.

**Results:**

Medicare beneficiaries with HAP were 2.8 times more likely to die within 90 days compared with matched beneficiaries who did not develop HAP. Among those who survived, beneficiaries with HAP spent 6.6 more days in the hospital (69%) and cost the Medicare program an average of $14,487 (24%) more per episode of care across initial inpatient and postdischarge services.

**Conclusions:**

The findings of higher mortality and cost among Medicare beneficiaries who develop HAP suggest that HAP prevention should be prioritized as a patient safety and quality initiative for the Medicare program.

Hospital-acquired pneumonia (HAP) is one of the most common healthcare-associated infections (HAIs) and represents a serious patient safety and quality of care issue.^1^ Patients coming to the hospital for treatment of other conditions may get HAP either associated with mechanical ventilation^1^ ventilator-associated pneumonia (VAP) or without ventilation^2^ nonventilator hospital-acquired pneumonia (NVHAP). Estimates indicate that approximately one-third of HAP cases occur in ventilated patients and two-thirds are NVHAP.^1,2^ The harm and significant costs of VAP have been well studied for decades. VAP is associated with a mortality rate of up to 17%, increased length of stay (LOS) of 6–25 days, and significant additional healthcare costs of $12,000–$40,000 per hospital admission.^3^ Even though NVHAP accounts for most HAP, the quality and quantity of research on NVHAP lags far behind research on VAP. However, a recent study looked at >6 million hospital admissions and found that NVHAP was attributed to 1 in 14 hospital deaths. In addition, patients who acquire NVHAP are often discharged to skilled nursing facilities; 8% are discharged to hospice versus 1.4% of the general hospital population, further adding to patient harm and associated cost.^4^ Overall, an emerging body of evidence indicates that HAP, and specifically NVHAP, is associated with increased hospital length of stay (LOS), need for intensive care, antibiotic use, incidence of sepsis, morbidity and mortality, 30-day hospital readmission rates, and higher overall healthcare costs.^4–9^

Despite the negative impact HAP places on patients and the healthcare system, to date, no acute inpatient hospital quality program implemented by Medicare includes measures to prevent NVHAP, and VAP is part of the ventilator-associated events prevention bundle that is recommended but not required.^10^ Given that HAP is the most common HAI, HAP prevention is of paramount importance for US healthcare, especially in the acute-care setting.

The objectives of this study were (1) to assess the impact of HAP among Medicare beneficiaries, and (2) within the limits of available data, to understand the individual impact VAP and NVHAP, compared to overall HAP, on the Medicare program.

## Methods

### Data set

The fiscal year 2019 (FY19) national hospital MedPAR claims data were used to estimate the cost of HAP to the Medicare program and its beneficiaries. For all other analyses, we used the 2019 calendar year (CY19) Medicare 5% Standard Analytic Files (SAF) for inpatient (including for inpatient rehabilitation and long-term hospitalizations), outpatient hospital, carrier (including physician service billing, laboratory billing, and ambulatory surgery center billing), skilled nursing facility, and home health data. We did not include outpatient drugs paid for under the Medicare Part D program. Cases were defined as Medicare beneficiaries with HAP using the *International Classification of Diseases, Tenth Revision, Clinical Modification* (ICD-10-CM) codes on inpatient claims. Cases were included if the hospital billed any pneumonia code (NVHAP J12-J18 or VAP J95.851) and identified as not present on admission. VAP cases were any HAP cases assigned to a ventilator diagnosis-related group (DRG) and NVHAP was the residual.

Beneficiaries who were not continuously enrolled in Medicare Part A and B were completely excluded from the study population. Among those who were continuously enrolled, the patients who died and those who had Medicare due to ESRD were excluded from some of the analyses. Patients who were not continuously enrolled in Medicare Part A and B were excluded due to the nonavailability of their full claims history during the nonenrolled months. Patients who died or were Medicare eligible by virtue of ESRD were excluded from the control and case groups cost and LOS comparison. This decision was made due to the potential impact of end-of-life care on hospital LOS as well as the high cost of healthcare associated with end-of-life care and ESRD.

### Case (HAP) versus control (non-HAP) DRGs

Beneficiaries who did not have HAP during 2019 were used as a control group. To control for possible variations in cost due solely to differences in patient health status, matched control-group beneficiaries were required to have been assigned to the same DRG for their initial hospitalization as study beneficiaries. A DRG is a case-mix system designed to categorize patients with similar clinical diagnoses to determine payor reimbursement rates.^11^ This DRG matching resulted in beneficiaries being matched by the ultimate severity of their hospital stay rather than by their initial reason for hospitalization, an approach that accounts for severity consistent with Medicare policy. Case patients were also matched with control patients on the variables of age, sex, and race. When multiple controls were identified for a case patient, the control patients included in the study were randomly selected from those identified. The matching criteria did not consider specific pneumonia type codes due to the volume of unspecified pneumonia type coding and the low utilization rate for the J95.851 code. Table 1 describes the case group and control groups.

**Table 1.** Study and Control Population Demographics

	After Matching			
	Case (HAP)	%	Control (Non-HAP)	%
Inpatient hospitalization	2,457	100.0	2,457	100.0
**Age**				
≤64 y	559	22.8	559	22.8
65–74 y	890	36.2	890	36.2
75–84 y	698	28.4	698	28.4
≥85 y	310	12.6	310	12.6
**Sex**				
Male	1,265	51.5	1,265	51.5
Female	1,192	48.5	1,192	48.5
**Race**				
White	2,010	81.8	2,010	81.8
Black	295	12.0	295	12.0
Other	152	6.2	152	6.2

Note. HAP, hospital-acquired pneumonia.

Source: Analysis of CY 2019 Medicare Standard Analytic Files.

### Cost comparative analysis

During the initial hospital stay, case patients and control patients were compared regarding hospital LOS, estimated hospital cost, and total Medicare payments. In the postacute periods of 30, 60, and 90 days following hospital discharge, case patients and control patients were compared regarding Medicare payments for the services received in each of the 3 periods.

As an additional analysis, the cost of a patient’s stay to the hospital (using charges reduced to cost based on data in the cost report), were determined using the traditional CMS method of using charges reduced to cost, based on data in the cost report. In all cases, Medicare payments included the beneficiaries’ coinsurance responsibility regardless of the beneficiaries’ ability to pay.

Costs after the initial hospitalization for both the case and control groups were measured in 30-, 60-, and 90-day periods. Payments were calculated for the following categories of Medicare services: (1) inpatient hospitalizations (short-term acute, long-term acute, and inpatient rehabilitation) after the initial stay; (2) outpatient hospital care; (3) physician care, including at ambulatory surgical centers and hospitals, and laboratory billing; (4) skilled nursing facility care; and (5) home health care. To eliminate confounding in the analysis, in the 90-day cost, we controlled for extreme differences in cost due to end-of-life care including ESRD because it would bias the cost substantially compared to any other type of measured condition.

### Mortality analysis

As previously described, because the control versus case groups used for LOS and cost comparisons excluded patients who died or were enrolled in Medicare for ESRD, to compare mortality, data on all beneficiaries without HAP were used for mortality comparison. This approach resulted in a larger sample size than for the sample size used for the general LOS and cost analyses. The 30-, 60-, and 90-day mortality rates were calculated using all beneficiaries who remained enrolled in Medicare for either 90 days following initial hospitalization discharge or until their death, whichever occurred first.

### Analysis of cost to the Medicare program and its beneficiaries

We also estimated the cost of HAP to the Medicare program and its beneficiaries. The cost estimate did not include expenditures for the Medicare Advantage program, for which CMS does not provide sufficient data for such an analysis. The number of HAP cases experienced by Medicare fee-for-service beneficiaries was estimated using 2019 MedPAR claims data, applying the same algorithm as described above. MedPAR consolidates inpatient hospital claims data from the National Claims History (NCH) files into a single hospital-stay–level record.

### Statistical analysis

Paired *t* tests were used to determine statistically significant differences in hospital cost of care (total, inpatient hospital, outpatient hospital, physician and lab services, skilled nursing facility, home health) and length of stay during various timeframes (initial hospitalization, 30 days after discharge, 60 days after discharge, 90 days after discharge). The cost estimate analysis was a point estimate conducted applying the costs calculated in the 90-day analysis and applying that savings rate to the full universe of HAP cases in the MedPAR file (which contains 100% of Medicare Part A discharges in the federal fiscal year.) The mortality analysis was an average rate of mortality in the unmatched populations.

### Human-subject research

The CMS Limited Data Set Standard Analytic Files (SAF), also known as Medicare claims files, are prepared with the intent of making them available to the public for use in healthcare research. The publicly available data are not individually identifiable; therefore, their use does not require human-subject research review.

## Results

For the major outcome of cost, Medicare payments were higher for beneficiaries with HAP for their initial hospitalization, as well as for subsequent hospitalizations (including for inpatient rehabilitation and long-term hospitalizations) and other services provided in the 90-day postdischarge period (Table 2).

**Table 2.** Initial Hospitalization and Post-Acute Care Analysis for Case and Control Groups

Variable	Case (HAP)		Control (Non-HAP)			
	Mean, $^a^	SD, $^a^	Mean $^a^	SD, $^a^	*P* Value^b^	% Difference
**Initial hospitalization**						
Inpatient total payments	43,960	61,494	33,767	39,334	<.0001	30
Length of stay, d	16.2	14.4	9.6	10.3	<.0001	69
**30 d after discharge**						
Total	12,835	23,057	11,238	19,998	.0092	14
Inpatient hospital	6,270	19,772	4,990	16,138	.0135	26
Outpatient hospital	820	2,291	1,064	2,536	.0002	−23
Physician and lab services	2,190	2,659	1,970	3,186	.0059	11
Skilled nursing facility	2,245	8,213	2,081	8,215	.4810	8
Home health	1,309	2,391	1,131	2,283	.0071	16
**60 d after discharge**						
Total	22,909	38,113	19,686	29,225	.0007	16
Inpatient hospital	11,200	32,093	8,749	21,931	.0017	28
Outpatient hospital	1,779	4,006	2,212	4,285	.0001	−20
Physician and lab services	3,814	4,741	3,441	5,670	.0085	11
Skilled nursing facility	4,305	11,800	3,732	11,806	.0817	15
Home health	1,811	2,857	1,553	2,690	.0010	17
**90 d after discharge**						
Total	30,695	46,606	26,401	35,696	.0002	16
Inpatient	15,186	37,581	11,711	25,518	.0001	30
Outpatient	2,767	5,960	3,437	6,489	<.0001	−19
Physician and lab services	5,235	6,559	4,714	7,766	.0076	11
Skilled nursing facility	5,378	13,678	4,734	13,429	.0809	14
Home health	2,129	3,091	1,805	2,946	.0001	18

Note. HAP, hospital-acquired pneumonia.

a Units unless otherwise specified.

b Paired *t* test.

Source: Analysis of CY 2019 Medicare Standard Analytic Files.

### Initial hospitalization

The increased cost of the initial hospitalization was driven, in part, by group differences in hospital length of stay (LOS). Case patients had a mean hospital LOS of 16.2 days, which was 69% longer than the matched control group LOS of 9.6 days. Costs for both beneficiaries and the hospital were higher for the case patients: $10,193 (30%) more for the individual beneficiary under the Medicare payment formula and $20,561 (63%) more per case for the hospital (*P* = .001) (Table 2).

Additionally, when VAP and NVHAP case patients were analyzed separately and compared to their matched controls, LOS was 7.4 days longer for VAP cases and 6.5 days longer for NVHAP cases. Medicare payments for the initial hospitalization were 31% higher for VAP cases, compared to the control group ($114,825 vs $87,941) and were 30% higher for NVHAP cases ($35,029 vs $26,939). Total estimated costs to the hospital for the inpatient stay (using charges reduced to cost based on data in the cost report) were also higher for the case versus control groups: VAP ($121,374 vs $85,141) and NVHAP ($44,662 vs $26,076). From the hospital’s perspective, there was a statistically significant difference between hospital costs and Medicare payments for VAP and NVHAP was $6,549 and $9,633, respectively (Table 3).

**Table 3.** Initial Hospitalization—Medicare Payment and Estimated Hospital Cost for All HAP, VAP, and NVHAP

Variable	All HAP (Mean)	VAP (Mean)	NVHAP (Mean)
Inpatient total payments Medicare payment	$43,960	$114,825	$35,029
Medicare estimated hospital cost	$53,248	$121,374	$44,662
Difference	($9,288)	($6,549)	($9,633)

Note. HAP, hospital-acquired pneumonia; VAP, ventilator-associated pneumonia; NVHAP, nonventilator hospital-acquired pneumonia.

Source: Analysis of CY 2019 Medicare Standard Analytic Files.

### Postacute hospital services analysis

**Figure 1.**
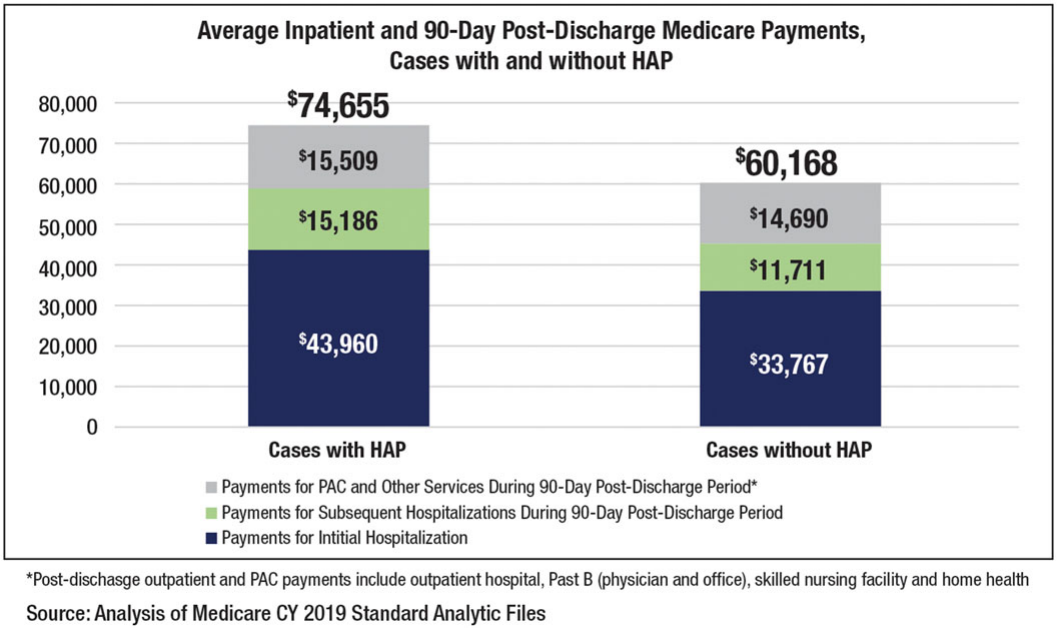
Mean total payments during initial hospitalizations and 90-day postdischarge payments for Medicare beneficiaries with hospital-acquired pneumonia (HAP) and matched comparison group.

Most previous analyses have not considered utilization of healthcare service in the postacute hospital phase of the episode, which can add significant expense to total cost of care. Total Medicare payments were compared between the case and control groups overall and at 30, 60, and 90 days after acute care in each of the 4 categories: outpatient hospital, physician and laboratory services, skilled nursing facility, and home health (Fig. 1).

Total Medicare payments were 14%–16% higher for beneficiaries with HAP compared to control-group patients at 30, 60, and 90 days after discharge (*P* < .01). At the 90-day mark, beneficiaries with HAP were $4,294 (16%) more expensive to treat per case versus control-group patients. Cases with HAP had statistically substantially higher payments for subsequent hospitalizations, physician and clinic services, laboratory services, and home health care in each postacute care period. In one cost category, outpatient hospital care, the control group had higher payments, possibly due to earlier discharge and a lower use of inpatient care (Table 2).

### Mortality analysis

Mortality was substantially higher for case versus control at each of the 30-, 60-, and 90-day postacute care periods (Table 4). Overall, case patients were 2.8 times more likely to die within 90 days compared to the control.

**Table 4.** Mortality Analysis

Postdischarge Mortality	All Cases			
	Case (HAP)	%	Control (Non-HAP)	%
Initial hospitalization	4,660	100.0	531,353	100.0
**Mortality**				
Died Within 30 d	1,440	30.9	46,019	8.7
Died Within 60 d	1,668	35.8	62,302	11.7
Died Within 90 d	1,817	39.0	74,185	14.0

Note. HAP, hospital-acquired pneumonia.

Source: Analysis of CY 2019 Medicare Standard Analytic Files.

### Fiscal impact analysis

Using 2019 MedPAR claims data, we located 140,911 Medicare fee-for-services beneficiary cases. Given the observed cost differential of $14,487 per case (including higher payments in the initial hospitalization and in the 90 days after discharge), we estimate that the cost to the Medicare program and its beneficiaries for patients who get HAP is $2.04 billion annually.

## Discussion

This analysis of Medicare claims data underscores the importance of HAP by quantifying the extent to which HAP is a high-volume, high-cost hospital-acquired condition with implications for quality of care and health outcomes for the Medicare program and its beneficiaries. Based on analysis of CY2019 fee-for-service Medicare claims data, beneficiaries with HAP spent almost 1 week longer in the hospital than similar beneficiaries without HAP. These patients have a greater need for postacute care services and have higher rates of mortality. HAP is also expensive, costing the Medicare program an average of $14,487 (24%) more per case across inpatient and 90-day postdischarge services compared with Medicare beneficiaries with the comparative acuity who did not get HAP. Furthermore, our results suggest that HAP leads to an estimated $2.04 billion annually in increased costs for the Medicare program and its beneficiaries.

Given the cost and patient harm associated with HAP, we believe that it is time for the Centers for Medicare & Medicaid Services (CMS) to include NVHAP in their required HAI reporting programs, especially given that most hospitals do not track their cases or engage in active prevention.^5,12^ Patient safety organizations have begun to address NVHAP. For example, the patient safety organization ECRI included NVHAP as one of its top 10 patient safety concerns for 2022.^13^ The Joint Commission issued an NVHAP Safety Alert.^14^ The National Organization for NVHAP Prevention (NOHAP) and The Joint Commission worked with several other safety organizations to publish a call to action to address this common and largely preventable HAI, which the NOHAP group estimates occurs in 1 in every 100 hospitalized patients.^5^

Examples of successful locally driven initiatives that have reduced HAP and NVHAP include the VA, Kaiser Permanente, and Sutter Health. The VA’s successful national campaign to reduce NVHAP by implementing an oral-care regimen, Hospital-acquired Pneumonia Prevention by Engaging Nurses (HAPPEN), which started in 1 facility in 2016 and has expanded to all VA hospitals nationally. VA hospitals that have implemented the program report a decrease in pneumonia rates of 40%–60%.^15^ Kaiser Permanente Northern California implemented an initiative to prevent NVHAP for high-risk patients and reduced the occurrence of NVHAP, antibiotic usage, as well as mortality from NVHAP.^16^ The Sutter Health NVHAP prevention initiative led to a significant reduction in the incidence of NVHAP, with results sustained over 4 years.^17^

The recent proliferation of guidelines for prevention coupled with successful reductions in HAP at major health systems who engage in prevention show that HAP can be diagnosed and prevented.^18^ Most recently, SHEA (Society for Healthcare Epidemiology of America), APIC Association for Professionals in Infection Control and Epidemiology), and IDSA (Infectious Diseases Society of America) released updated Practice Recommendations to prevent HAP that included NVHAP for the first time.^18^ Fortunately, recent research and structures are now available for hospitals to monitor NVHAP more easily and launch prevention efforts. Research by Klompas et al^4^ demonstrated that NVHAP surveillance can be accurately and efficiently extracted directly from the electronic health record. Structured methods for capturing implementation of best HAP prevention practices such as oral care are also being considered. For example, internationally, the Systematized Nomenclature of Medicine—Clinical Terms (SNOMED CT) recently added the Department of Veterans’ Affairs (VA) oral care structure to its system. SNOMED nomenclature is a required part of the electronic health record in the United States; therefore, hospitals will have access to the data collection process.^19^

This research has shown significant impact regarding both patient safety and cost to the Medicare Program. The Medicare program has a range of policy options to address this patient safety concern. As previously mentioned, we believe that CMS and Medicare should include both VAP and NVHAP in existing, or reformed, acute inpatient hospital quality programs, starting with specific reporting requirements for each. CMS should engage in additional collaboration with the CDC, specifically to support the development and advancement of valid and reliable quality measures that are feasible to collect and that address the epidemiology of HAP in hospitals. CMS could also build on its work to support collaborative efforts to share best practices and quality measure practice improvements. Continuing CMS partnerships with existing patient safety organizations developed during the COVID-19 pandemic along with learning from the research underway at the Veterans’ Health Administration could also support methods and strategies for addressing HAP issues in the Medicare program. The CMS Innovation Center should also consider including HAP in its innovation models.

This study had several limitations. These analyses were conducted using administrative claims data, which do not contain the same clinical information as the medical record and which rely upon the accuracy of hospital coding practices from information in the medical records onto claims forms. Using this strategy did not enable us to account for any regional differences that may have existed in LOS and costs. Pneumonia is a complex clinical condition, and its coding and reporting are associated with substantial challenges for achieving accuracy, consistent diagnosis, and meeting reporting requirements. Challenges include distinguishing NVHAP from VAP and a reliance on DRG coding and day of event. This information is also subject to variation among hospitals. Despite these limitations, claims data continue to be an important source of healthcare information, which is used for both required reporting and payments to hospitals.

Because the current CMS and CDC regulations do not require reporting on HAP, NVHAP, VAP or VAE, the degree to which HAP prevention strategies were applied to beneficiaries in the study population is unknown, and the proportion of the identified cases of HAP that are preventable is also unknown. However, recent research supports that overall, little prevention is being undertaken, especially for NVHAP.^5,12^

In conclusion, as the federal government continues to consider how best to rebuild our healthcare system, a serious commitment to HAP prevention will likely yield significant benefits to both the Medicare program and the US healthcare system overall. As policy makers continue to look for ways to improve patient outcomes and patient safety within the Medicare program, HAP and especially NVHAP, represent a substantial opportunity to positively impact a high-risk, high-cost, and largely preventable condition.
